# Do Years of Running Experience Influence the Motivations of Amateur Marathon Athletes?

**DOI:** 10.3390/ijerph17020585

**Published:** 2020-01-16

**Authors:** Ewa Malchrowicz-Mośko, François Gravelle, Agata Dąbrowska, Patxi León-Guereño

**Affiliations:** 1 Faculty of Sports Sciences, Poznan University of Physical Education, 61-871 Poznan, Poland; zagata.pl@gmail.com; 2 Faculty of Health Sciences, University of Ottawa, Ottawa, ON K1N 6N5 ON, Canada; fgravel@uottawa.ca; 3 Faculty of Psychology and Education, University of Deusto, 5001 San Sebastian, Spain; patxi.leon@deusto.es

**Keywords:** running, marathon, motivations, amateur runner, experience

## Abstract

The aim of the study was to investigate if years of running experience influence the motivations of marathon athletes. An empirical study was conducted during the last (20th) PKO Poznan Marathon, one of the largest and most popular mass running events in Poland, which was held in Poznan (Poland) in October 2019. A total of 493 marathon runners (29% of whom were female, and 71% of whom were male) took part in the cross-sectional study, which used the diagnostic survey method. The questionnaire employed the division of motives from the motivation of marathoners scale (MOMS) by Masters et al., adapted to the Polish language by Dybala. Running motivations have already been analysed for variables such as age, gender and place of residence, but there is a research gap regarding existing research, as the relationship between motivations and running experience has not yet been studied. One-way analysis of variance for independent samples was used to verify statistical hypotheses. Prior to making the relevant calculations, the assumption of homogeneity of variance was checked via Levene’s test. Variances were assessed with an F-test, and if they were unequal, Welch’s correction was applied. Eta squared (η^2^) was used as a measure of effect size. The calculations carried out showed that running experience was not a statistically significant factor in the motivations of runners taking part in a marathon.

## 1. Introduction

In recent years, marathon running has become a mass sport. It seems worth asking why so many runners are motivated to undertake such a gruelling activity. There are many studies on the typology of runners and the sociodemographic profiles of participants in mass running events—half-marathons, marathons, ultra-marathons, triathlons or ultra-triathlons—and their motivational structures. Running motivations have already been analysed for variables such as age, gender and place of residence [1,2,3,4,5,6,7,8,9,10,11,12,13]. Poczta et al., (2018) investigated age-related motivations in half-marathon participation. The most significant difference they found between older and younger runners was that older people were more often focused on social aspects and contact with others, while younger people were more focused on results [14]. According to Ogles et al., younger runners are more often motivated by personal goal achievement, while older runners are more motivated by life meaning, health and weight orientation or by affiliation with other runners [15]. Ferrer et al., (2015) discovered that older runners are more motivated to train by physical factors than younger ultramarathon runners [16]. Saayman et al. also found a statistically significant motivational difference in age among triathletes [17].

In terms of gender, vast research has been conducted on motivational differences in mass running [18]. According to Ogles and Masters, the most common motivations for running among women include social needs and good physical condition, while men are more likely to compete and achieve success [19]. Summers et al. indicated that female runners adduce opportunities to meet new people and old friends more often than male runners [20]. Malchrowicz-Mośko and Poczta found that the most significant differences between male and female motivations were the desire to get away from everyday life and the prevailing fashion for mass running, which often turned out to be more important for women than for men. While the desire to win was not equally important for both genders, the need to experience strong emotions during the race, the need to feel integrated and unified with other runners and the desire to test themselves were equally important [21]. Yates et al. identified some similarities between anorexic women and men who were “obligatory runners.” They also claimed that female runners tended to be evaluated by their physical attractiveness, weight and fitness, while male runners tend to be evaluated on their physical strength and effectiveness [22,23,24]. Recent studies on ultra-marathons have indicated that whereas the rivalry factor has always been more important for men, it has also gained importance among women [25]. Recently Nikolaidis et al., (2019) partially confirmed that female and male marathon runners differ in their motivations [26]. Smith (2010) investigated the motivations of female elite triathletes [27], and Fernandez-Lopez et al. [28] examined the relationship between sex and motivation in triathletes. Men and women competing at the international level in triathlons were found to have similar motivational profiles.

In terms of place of residence, Poczta and Malchrowicz-Mośko reported on the motivational differences regarding doing a half-marathon among two groups of runners—those living in rural areas and those living in big cities. Test results indicated that the difference between runners from rural and urban areas lies in the motivations connected with sensation-seeking orientation. Rural residents more often claimed that the most important motivation for them was the need to experience strong sensations and emotions related to running in mass sporting events [29]. Motivations of urban runners were also studied in various countries; e.g., in Chile [30]. Parra-Camacho et al., (2019) investigated sporting habits of urban runners according to their motivation [31].

There are also studies on the motivational differences between local runners and sports tourists [32,33], and some papers have been published on university students and female local runners and female sports tourists. Test results indicated that local runners and sports tourists had similar motivations to run in mass events, albeit with some statistically significant differences [34,35].

Some experienced runners have recently become ultra-runners or even triathletes. Since these are new sports, there is a paucity of studies in the area of motivations, but as the triathlon is an endurance sport, the reasons or motivations to compete could be linked to research in other endurance sports. Lovett et al., (2018) checked the motives for participating in triathlon competitions [36]. Croft et al. investigated the motivation levels between non-elite and elite triathletes. The instrument used was a modified version of the motivation of marathoners scale (MOMS), which found rivalry and goal achievements to be the main motivations for competing [37].

Hanson et al., (2015) checked motivational differences between half, full and ultra-marathoners using running distance as a variable. Compared to half and full marathoners, ultra-marathoners scored lower on weight concern and health orientation and higher on life meaning. Full marathoners scored higher than ultra-marathoners on personal goal achievement. Ultra-marathoners declared more intrinsic motives for running than the other distance groups [38]. According to Waśkiewicz et al., ultra-runners have greater orientation to life meaning and social affiliation than to self-esteem, personal goal achievement and weight concerns [39].

Motivational aspects have also been analysed depending on context; hence, comparing participants in traditional mass sports events (e.g., half-marathons and marathons) and non-traditional events (e.g., obstacle races) to find whether different events may attract different individual motivations toward participation. Results showed that participants motivation was statistically different in seven out of nine dimensions of the MOMS scale [40].

Ogles and Masters grouped athletes’ motivations into five different dimensions through a cluster analysis, which at the same time showed statistical differences according to runners’ training patterns, running experience and demographic variables [2] in a study carried out sixteen years ago. Due to the change in participants’ motivations in the last years, it may be worth re-analysing athletes’ motivations for participation in mass running events [40]. It can be said that very little is known about the relationship between years of training and athletes’ participation motivations. Moreover, the update of the MOMS research tool has been presented [41]; however, it has never been adapted in many countries. Poland, for instance.

As the literature review shows, the relationship between motivations and running experience has not been analysed to date. In order to bridge this research gap and improve the understanding of amateur runners’ participation in mass running events, the aim of this study was to identify running motivations depending on years of training. Consequently, it was hypothesised that different running motivations would be found among marathon athletes according to their years of running experience.

## 2. Materials and Methods

### 2.1. Participants and Design

This cross-sectional study involved 493 amateur runners, 29% of whom (*n* = 144) were female, and 71% of whom (*n* = 349) were male. The data were chosen randomly from among the participants in the 20th PKO Poznan Marathon, which took place in October 2019 in Poznan, Poland. We tried to make the sample selection in a way that ensured the best possible representativeness of the results obtained. Table 1 shows the descriptive statistics of participants, in which the runners were divided into 4 groups by gender, age and years of running experience (I have run for less than 3 years; I have run for 3–5 years; I have run for 5–10 years; I have run for over 10 years).

### 2.2. Questionnaire

The diagnostic survey was conducted using the MOMS [42]. The questionnaire research tool was received from one of the authors—B. Ogles—in September 2019. The Polish and English-language versions of the questionnaire were sent to the runners by the organising committee of the PKO Poznan Marathon at the request of the authors. The Polish adaptation of the MOMS proposed by M. Dybala in 2013 [43] was used. The MOMS contains 56 motives rated on a 7-point Likert-type scale in terms of the importance of motives for a runner (1 = minimum and 7 = maximum). The MOMS groups all items into 9 main theme groups or dimensions: health orientation, weight concern, personal goal achievement, competition, recognition, affiliation, psychological coping, life meaning and self-esteem. Participants were treated in accordance with the guidelines of the Publication Manual of the American Psychological Association [44] regarding consent and anonymity. Athletes were contacted via email and provided with detailed information about the study. The survey was created using Google Docs technology [45].

### 2.3. Data Analysis

One-way analysis of variance for independent samples was used to verify statistical hypotheses. After checking the assumption of homogeneity of variance via Levene’s test, the calculations were made. Variances were assessed with an F-test, and if they were unequal, Welch’s correction was applied. Eta squared (η^2^) was used as a measure of effect size. The results were statistically significant at the *p* < 0.05 level. Statistica 10.0 software (Statsoft Inc., Cracow, Poland, 2011) was used to perform the analysis.

## 3. Results

Table 2 shows the average and standard deviation of the sample in each of the nine dimensions that were analysed through the MOMS. Health orientation (5.46 ± 1.17), personal goal achievement (5.05 ± 1.37) and self-esteem (4.72 ± 1.39) scored the highest, and recognition (2.85 ± 1.37) and competition (2.01 ± 1.50) scored the lowest as motives for participation. The rest of the theme groups were between the opposite poles: Psychological coping (4.26 ± 1.36). Weight concerns (4.14 ± 1.7). Life meaning (3.97 ± 1.49) and Affiliation (3.51 ± 1.61).

**Figure 1:**
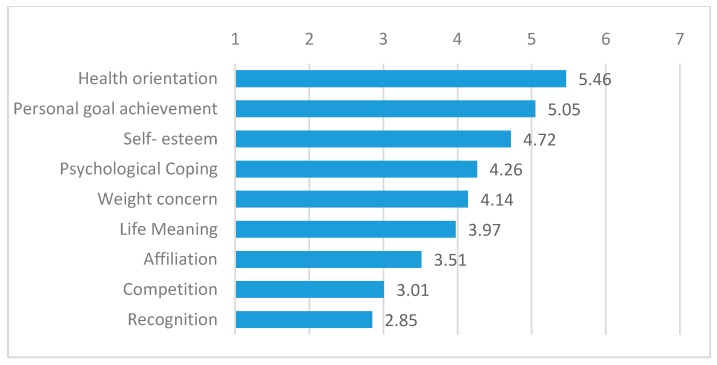
Averages in descending order. Source: developed by the authors.

Figure 1 shows the average motivational scores for the nine dimensions of the MOMS in a descending order. General health orientation was the highest motivation to participate (5.46), followed by personal goal orientation (5.05) and self-esteem (4.72). Psychological coping (4.26), weight concern (4.14) and life meaning (3.97) showed slightly lower values, closer to four. Affiliation (3.51), competition (3.01) and recognition (2.85) had the lowest scores among participants as motivations for practising this sport.

Table 3 shows the associations among the four groups with different running experience (1 = less than 3 years; 2 = between 3 and 5 years; 3 = 5 to 10 years; and 4 = more than 10 years). No statistically significant differences were found for any of the nine participation motivations according to the years of running experience. The weight concern dimension shows results close to statistical significance (0.061).

**Figure A1:**
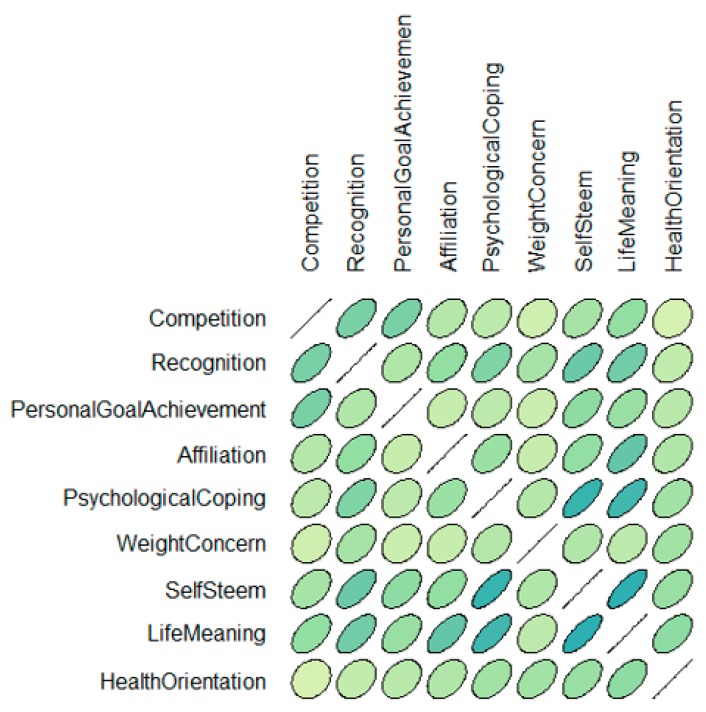
Cronbach’s alpha. Source: developed by the authors.

The analysis of the significance of differences between the four groups (according to years of running training) in relation to all the 56 MOMS motives and Cronbach’s alpha have been presented in Appendix A (Figure A1 and Table A1).

## 4. Discussion

Recently, running events have enjoyed growing interest among researchers. They have been analysed as a stimulator of touristic development [46]. Not only motivations of runners [47], but also running supporters have been examined [48], as has the impact of running events on the local community [49]. Malchrowicz-Mośko et al., (2018) also determined what impact achieving a self-set sports goal had on the level of satisfaction with running in a half-marathon. They checked whether runners who did not set themselves any sports goals and simply ran for pleasure achieved the same level of satisfaction as runners who set themselves a demanding sports goal and achieved it, and found that participants who did not set a sports goal experienced the same degree of satisfaction as runners who achieved their ambitious sports goal [50]. Understanding the motivations of runners is important from the point of view of managing mass sport and promoting a healthy lifestyle, so the aim of our study was to analyse amateur athletes’ motivations for participation in marathons according to their years of running experience. In order to verify the hypothesis about a statistically significant impact of running experience on the scales of the MOMS questionnaire, a one-way analysis of variance was performed for independent samples. The results obtained through the MOMS showed that amateur runners’ main participation motives were related to general health, personal goal achievement and self-esteem. Partially consistent with these results, Ogles and Masters found that one of the main motivations for participation in mass running events among younger marathoners was personal goal achievement [15,38]; in contrast, physical health was not found to be one of the main participation motives among young athletes, but it was one of the main motives for participation among elder runners. Their research showed a clear orientation toward social needs for women and toward performance among men [19], the latter being in line with our results.

This study shows how the main participation motivations were general health orientation and personal goal achievement, in line with previous research, where good health and testing themselves to achieve set goals were found to be the main participation motives among marathoners [29,38]. Malchrowicz-Mosko and Poczta analysed half-marathon runners’ motives for participation and obtained results partially in contrast with our research. They showed that participants’ main motivations were not related to performance or personal achievement, and found the most important motives for participation to be related to the need to experience strong emotions and to social motives [21]. These findings are in contrast with the motivational aspects of ultra-marathoners, who showed a more intrinsically motivated orientation. The main reasons for them to take part in a race were related to social motives [38], whereas affiliation and social recognition had the lowest scores in our research. Apart from ultra-marathoners, athletes’ motives for participation have been analysed in other endurance sports, such as cycling, for which the main participation motives were health-related in women and performance-related in men [51], partly in line with our results.

Athletes’ participation motivations have also been assessed according to social context [29] and type of event; i.e., traditional versus non-traditional endurance events. Significant differences were found in seven out of the nine dimensions of the MOMS scale. This last study was consistent with our results and showed that the motivations for participation in traditional running events such as marathons were health-related and associated with personal goal achievement, while non-traditional event endurance athletes showed greater emphasis on social participation motivations [40], in contrast with our results.

Ogles and Masters described the following five definable groups of people motivated to participate in a marathon: running enthusiasts, lifestyle managers, personal goal achievers, personal accomplishers and competitive achievers. These authors found significant differences among the previous clusters according to training patterns, demographic variables and running experience [2], this last one being in contrast with our results, since no significant differences were found in any of the nine dimensions of the MOMS according to marathoners’ running experience.

In 1995, Masters and Ogles investigated the motivation characteristics of marathon runners who varied in their participation experience. The most experienced veterans, who had participated in more than three marathons, were motivated more by social and competitive reinforcements than by personal accomplishment or internal psychological rejuvenation. The mid-level experienced runners, after their second or third marathon, were primarily motivated by personal performance enhancement and psychological rewards. For the rookie marathon runners, self-esteem appeared to be a more important motivation than for the more experienced runners [42,52]. Twenty-five years later, our research about runners who vary in years of training does not confirm the study by Masters and Ogles.

## 5. Conclusions

The results of calculations do not give grounds for adopting alternative hypotheses. Statistically non-significant results were obtained for all nine scales of the questionnaire. The differences between the means were non-significant, and the eta squared value (η^2^) indicated a very small effect size. Even though a previous study had shown differences according to years of running experience [2], these results have not been analysed according to the MOMS dimensions yet. Due to the changes in participants’ motivations in recent years, it has been suggested that athletes’ motives to participate should be re-analysed [40]. Therefore, the relationship between participation motives and years of experience remains unclear, since the calculations carried out did not show that the running experience was a statistically significant factor for differentiating the motivations of runners taking part in a marathon.

Further research is needed to gain a better understanding of this issue and obtain further insight into the nature of marathoners’ participation in connection with their running experience. A good method would be a longitudinal study. This study has some limitations, since the cross-sectional research did not allow causal inferences to be made among the variables studied. To conclude, in future research, gathering the data at two different times would provide a wider dataset and enhanced knowledge [53] about whether the years of experience of athletes are related to, or the extent to which they are may be related to, their participation motivations. Another interesting research line would be to analyse the differences between marathoners’ male and female experiences, since previous research [18,19] has shown significant differences in relation to the motivational variables according to gender. It would be also worth checking out other sociodemographic characteristics.

## Figures and Tables

**Table 1:** Descriptive characteristics of the respondents.

	*n*	%
**Gender**		
Women	144	29.21
Men	349	70.79
**Age**		
18 or under	5	1.01
19–25	46	9.33
26–35	166	33.67
36–50	252	51.12
51–70	24	4.87
**Years of running experience**		
less than 3	148	30.02
3–5	174	35.29
5–10	120	24.34
more than 10	51	10.34

Source: Developed by the authors.

**Table 2:** Basic descriptive statistics, *n =* 493.

Dimensions	Average	SD
Health orientation	5.46	1.27
Weight concern	4.14	1.70
Personal goal achievement	5.05	1.37
Competition	3.01	1.50
Recognition	2.85	1.37
Affiliation	3.51	1.61
Psychological Coping	4.26	1.36
Life Meaning	3.97	1.49
Self-esteem	4.72	1.39

Source: developed by the authors.

**Table 3:** Comparisons according to running experience.

Scale	Measure	Running Experience				F	*p*	η^2^
		Less Than 3 Years	3–5 Years	5–10 Years	More Than 10 Years			
Health orientation	M	5.28	5.55	5.61	5.38	1.86	0.135	0.011
	SD	1.40	1.18	1.19	1.28			
Weight concern	M	3.89	4.29	4.36	3.87	2.49	0.061	0.016
	SD	1.86	1.57	1.59	1.75			
Personal goal achievement	M	4.98	5.03	5.22	4.91	0.95	0.415	0.006
	SD	1.47	1.31	1.33	1.41			
Competition	M	2.82	3.06	3.23	2.85	1.88	0.131	0.011
	SD	1.52	1.46	1.51	1.55			
Recognition	M	2.85	2.89	2.82	2.76	0.15	0.929	0.001
	SD	1.38	1.38	1.26	1.53			
Affiliation	M	3.39	3.74	3.32	3.54	2.09	0.100	0.013
	SD	1.71	1.56	1.50	1.67			
Psychological Coping	M	4.17	4.29	4.39	4.13	0.81	0.490	0.005
	SD	1.35	1.34	1.31	1.54			
Life Meaning	M	3.93	4.02	4.02	3.83	0.31	0.822	0.002
	SD	1.60	1.46	1.39	1.54			
Self- esteem	M	4.78	4.78	4.70	4.39	1.11	0.346	0.007
	SD	1.42	1.36	1.37	1.47			

Source: developed by the authors. Groups two (3–5 years) and three (5–10 years) showed generally higher motivation in most of the dimensions than Groups one and four.

## Appendix A

- Health orientation: 0.8816112;
- Weight concern: 0.8799456;
- Personal goal achievement: 0.8841617;
- Competition: 0.8276396;
- Recognition: 0.8428978;
- Affiliation: 0.9085717;
- Psychological coping: 0.8824675;
- Life meaning: 0.8477674;
- Self-esteem: 0.8788929.

Below has been presented the analysis of the significance of differences between the four groups (according to years of running training) in relation to all the 56 MOMS motives.

The analysis does not provide grounds for adopting the hypothesis about a statistically significant impact of running experience on the validity of the motives of the MOMS questionnaire. However, some of the probability values obtained are slightly higher than the assumed significance level (*p* = 0.05), which means results at the level of the tendency toward significance.

**Table A1:** The analysis of the significance of differences between the four groups (according to years of running training) in relation to all the 56 MOMS items.

	Average	N	Standard Deviation	F	*p*
1. TO HELP CONTROL MY WEIGHT					
less than 3 years of running training	3.83	148	2.13	2.29	0.079
3–5	4.34	174	1.90		
5–10	4.38	120	1.82		
more than 10 years	4.04	51	1.97		
altogether	4.17	493	1.97		
2. TO COMPETE WITH OTHERS					
less than 3 years of running training	2.83	148	1.70	2.16	0.092
3–5	3.26	174	1.73		
5–10	3.29	120	1.75		
more than 10 years	3.22	51	1.88		
altogether	3.13	493	1.75		
3. TO EARN RESPECT OF PEERS					
less than 3 years of running training	2.26	148	1.59	0.30	0.823
3–5	2.36	174	1.63		
5–10	2.33	120	1.43		
more than 10 years	2.49	51	1.68		
altogether	2.34	493	1.57		
4. TO REDUCE MY WEIGHT					
less than 3 years of running training	3.15	148	2.16	2.17	0.091
3–5	3.53	174	2.06		
5–10	3.77	120	1.94		
more than 10 years	3.29	51	2.12		
altogether	3.45	493	2.07		
5. TO IMPROVE MY RUNNING SPEED					
less than 3 years of running training	4.39	148	1.86	1.38	0.248
3–5	4.61	174	1.66		
5–10	4.82	120	1.60		
more than 10 years	4.61	51	1.92		
altogether	4.59	493	1.74		
6. TO EARN THE RESPECT OF PEOPLE IN GENERAL					
less than 3 years of running training	2.47	148	1.71	0.60	0.618
3–5	2.69	174	1.76		
5–10	2.48	120	1.45		
more than 10 years	2.65	51	1.88		
altogether	2.57	493	1.69		
7. TO SOCIALIZE WITH OTHER RUNNERS					
less than 3 years of running training	3.40	148	2.00	1.91	0.127
3–5	3.86	174	1.93		
5–10	3.43	120	1.87		
more than 10 years	3.73	51	1.94		
altogether	3.60	493	1.94		
8. TO IMPROVE MY HEALTH					
less than 3 years of running training	5.76	148	1.51	1.07	0.365
3–5	5.99	174	1.11		
5–10	5.93	120	1.32		
more than 10 years	5.75	51	1.45		
altogether	5.88	493	1.33		
9. TO COMPETE WITH MYSELF					
less than 3 years of running training	6.18	148	1.49	0.01	0.999
3–5	6.19	174	1.34		
5–10	6.18	120	1.41		
more than 10 years	6.22	51	1.08		
altogether	6.19	493	1.38		
10. TO BECOME LESS ANXIOUS					
less than 3 years of running training	3.08	148	2.15	0.38	0.767
3–5	3.10	174	2.06		
5–10	3.33	120	2.10		
more than 10 years	3.12	51	2.31		
altogether	3.15	493	2.12		
11. TO IMPROVE MY SELF-ESTEEM					
less than 3 years of running training	4.77	148	2.16	1.96	0.119
3–5	4.65	174	2.04		
5–10	4.58	120	2.15		
more than 10 years	3.94	51	2.38		
altogether	4.60	493	2.14		
12. TO HAVE SOMETHING IN COMMON WITH OTHER PEOPLE					
less than 3 years of running training	3.30	148	2.07	1.07	0.362
3–5	3.35	174	1.93		
5–10	2.99	120	1.70		
more than 10 years	3.14	51	1.89		
altogether	3.23	493	1.92		
13. TO ADD A SENSE OF MEANING TO LIFE					
less than 3 years of running training	4.26	148	2.19	0.61	0.608
3–5	4.07	174	2.08		
5–10	4.18	120	1.99		
more than 10 years	3.82	51	2.12		
altogether	4.13	493	2.09		
14. TO PROLONG MY LIFE					
less than 3 years of running training	4.69	148	2.05	2.21	0.086
3–5	5.17	174	1.81		
5–10	5.16	120	1.81		
more than 10 years	4.82	51	2.00		
altogether	4.99	493	1.91		
15. TO BECOME LESS DEPRESSED					
less than 3 years of running training	3.89	148	2.37	0.19	0.900
3–5	3.99	174	2.19		
5–10	4.07	120	2.15		
more than 10 years	4.12	51	2.34		
altogether	3.99	493	2.25		
16. TO MEET PEOPLE					
less than 3 years of running training	3.84	148	2.14	1.27	0.283
3–5	4.18	174	1.88		
5–10	3.83	120	1.99		
more than 10 years	4.20	51	2.09		
altogether	3.99	493	2.01		
17. TO BECOME MORE PHYSICALLY FIT					
less than 3 years of running training	6.31	148	1.21	0.38	0.769
3–5	6.35	174	0.93		
5–10	6.43	120	0.89		
more than 10 years	6.41	51	0.85		
altogether	6.37	493	1.00		
18. TO DISTRACT MYSELF FROM DAILY WORRIES					
less than 3 years of running training	5.42	148	1.70	1.10	0.350
3–5	5.65	174	1.53		
5–10	5.63	120	1.54		
more than 10 years	5.27	51	1.90		
altogether	5.54	493	1.63		
19. TO MAKE MY FAMILY OR FRIENDS PROUD OF ME					
less than 3 years of running training	4.18	148	2.02	1.67	0.173
3–5	4.02	174	1.85		
5–10	3.78	120	1.85		
more than 10 years	3.59	51	1.96		
altogether	3.97	493	1.92		
20. TO MAKE MY LIFE MORE PURPOSEFUL					
less than 3 years of running training	4.76	148	1.96	1.06	0.367
3–5	4.74	174	1.82		
5–10	4.62	120	1.78		
more than 10 years	4.25	51	2.01		
altogether	4.67	493	1.87		
21. TO LOOK LEANER					
less than 3 years of running training	3.98	148	2.30	2.38	0.071
3–5	4.38	174	1.86		
5–10	4.47	120	1.86		
more than 10 years	3.76	51	2.11		
altogether	4.22	493	2.04		
22. TO TRY TO RUN FASTER					
less than 3 years of running training	4.73	148	1.94	0.48	0.694
3–5	4.79	174	1.73		
5–10	4.93	120	1.76		
more than 10 years	4.59	51	1.87		
altogether	4.78	493	1.81		
23. TO FEEL MORE CONFIDENT ABOUT MYSELF					
less than 3 years of running training	4.75	148	2.02	1.64	0.179
3–5	4.69	174	1.79		
5–10	4.58	120	1.75		
more than 10 years	4.10	51	2.06		
altogether	4.62	493	1.89		
24. TO PARTICIPATE WITH MY FAMILY OR FRIENDS					
less than 3 years of running training	3.46	148	2.02	1.93	0.124
3–5	3.89	174	1.97		
5–10	3.40	120	1.82		
more than 10 years	3.57	51	2.15		
altogether	3.61	493	1.97		
25. TO MAKE MYSELF FEEL WHOLE					
less than 3 years of running training	3.40	148	2.16	0.65	0.583
3–5	3.57	174	1.95		
5–10	3.31	120	1.95		
more than 10 years	3.20	51	2.00		
altogether	3.42	493	2.02		
26. TO REDUCE MY CHANCE OF HAVING A HEART ATTACK					
less than 3 years of running training	4.39	148	2.08	1.90	0.128
3–5	4.66	174	1.92		
5–10	4.94	120	1.87		
more than 10 years	4.45	51	1.89		
altogether	4.63	493	1.96		
27. TO MAKE MY LIFE MORE COMPLETE					
less than 3 years of running training	4.49	148	2.07	0.26	0.851
3–5	4.64	174	1.99		
5–10	4.54	120	1.97		
more than 10 years	4.39	51	1.59		
altogether	4.55	493	1.97		
28. TO IMPROVE MY MOOD					
less than 3 years of running training	5.53	148	1.45	0.14	0.933
3–5	5.57	174	1.41		
5–10	5.50	120	1.48		
more than 10 years	5.65	51	1.26		
altogether	5.55	493	1.42		
29. TO IMPROVE MY SENSE OF SELF-WORTH					
less than 3 years of running training	4.78	148	2.03	1.72	0.162
3–5	4.63	174	1.99		
5–10	4.52	120	1.94		
more than 10 years	4.06	51	2.17		
altogether	4.59	493	2.01		
30. TO SHARE A GROUP IDENTITY WITH OTHER RUNNERS					
less than 3 years of running training	3.47	148	2.04	1.99	0.115
3–5	3.87	174	1.99		
5–10	3.36	120	1.79		
more than 10 years	3.49	51	1.90		
altogether	3.58	493	1.95		
31. IT IS A POSITIVE EMOTIONAL EXPERIENCE					
less than 3 years of running training	5.71	148	1.53	1.34	0.263
3–5	5.97	174	1.23		
5–10	5.74	120	1.40		
more than 10 years	5.98	51	1.27		
altogether	5.84	493	1.37		
32. TO FEEL PROUD OF MYSELF					
less than 3 years of running training	5.82	148	1.69	1.61	0.185
3–5	5.57	174	1.69		
5–10	5.50	120	1.60		
more than 10 years	5.27	51	1.81		
altogether	5.60	493	1.69		
33. TO VISIT WITH FRIENDS					
less than 3 years of running training	2.85	148	1.91	1.96	0.119
3–5	3.31	174	1.87		
5–10	2.91	120	1.75		
more than 10 years	3.10	51	1.85		
altogether	3.05	493	1.86		
34. TO FEEL A SENSE OF ACHIEVEMENT					
less than 3 years of running training	3.65	148	2.01	0.93	0.426
3–5	3.79	174	2.01		
5–10	3.61	120	1.91		
more than 10 years	3.27	51	1.99		
altogether	3.65	493	1.98		
35. TO PUSH MYSELF BEYOND MY CURRENT LIMITS					
less than 3 years of running training	4.93	148	1.97	1.29	0.278
3–5	5.03	174	1.69		
5–10	5.28	120	1.72		
more than 10 years	4.75	51	2.05		
altogether	5.03	493	1.82		
36. TO HAVE TIME ALONE TO SORT THINGS OUT					
less than 3 years of running training	4.18	148	2.05	1.74	0.160
3–5	4.21	174	1.91		
5–10	4.56	120	1.63		
more than 10 years	3.98	51	1.93		
altogether	4.26	493	1.90		
37. TO STAY IN PHYSICAL CONDITION					
less than 3 years of running training	5.85	148	1.49	0.44	0.721
3–5	5.91	174	1.16		
5–10	6.03	120	1.17		
more than 10 years	5.96	51	1.02		
altogether	5.92	493	1.26		
38. TO CONCENTRATE ON MY THOUGHTS					
less than 3 years of running training	4.57	148	1.95	1.79	0.150
3–5	4.83	174	1.78		
5–10	4.94	120	1.65		
more than 10 years	4.35	51	1.93		
altogether	4.73	493	1.82		
39. TO SOLVE PROBLEMS					
less than 3 years of running training	3.87	148	2.13	1.03	0.379
3–5	4.01	174	1.98		
5–10	4.07	120	1.95		
more than 10 years	3.51	51	2.09		
altogether	3.93	493	2.03		
40. TO SEE HOW HIGH I CAN PLACE IN RACES					
less than 3 years of running training	3.32	148	1.96	0.70	0.550
3–5	3.55	174	1.88		
5–10	3.50	120	1.91		
more than 10 years	3.18	51	2.07		
altogether	3.43	493	1.93		
41. TO FEEL A SENSE OF BELONGING IN NATURE					
less than 3 years of running training	3.50	148	2.09	0.78	0.503
3–5	3.77	174	1.96		
5–10	3.84	120	1.89		
more than 10 years	3.76	51	2.06		
altogether	3.71	493	2.00		
42. TO STAY PHYSICALLY ATTRACTIVE					
less than 3 years of running training	4.59	148	1.96	1.56	0.198
3–5	4.91	174	1.68		
5–10	4.81	120	1.83		
more than 10 years	4.37	51	1.81		
altogether	4.73	493	1.82		
43. TO GET A FASTER TIME THAN MY FRIENDS					
less than 3 years of running training	2.73	148	1.94	1.41	0.238
3–5	2.84	174	1.80		
5–10	3.13	120	1.91		
more than 10 years	2.61	51	1.77		
altogether	2.86	493	1.87		
44. TO PREVENT ILLNESS					
less than 3 years of running training	4.69	148	2.04	2.14	0.096
3–5	5.19	174	1.72		
5–10	5.15	120	1.77		
more than 10 years	4.88	51	1.94		
altogether	5.00	493	1.86		
45. PEOPLE LOOK UP TO ME					
less than 3 years of running training	2.63	148	1.81	0.21	0.888
3–5	2.63	174	1.75		
5–10	2.56	120	1.63		
more than 10 years	2.43	51	1.73		
altogether	2.59	493	1.73		
46. TO SEE IF I CAN BEAT A CERTAIN TIME					
less than 3 years of running training	4.64	148	1.99	1.00	0.392
3–5	4.59	174	1.79		
5–10	4.93	120	1.84		
more than 10 years	4.51	51	1.83		
altogether	4.68	493	1.87		
47. TO BLOW OFF STEAM					
less than 3 years of running training	2.74	148	1.87	0.05	0.985
3–5	2.67	174	1.75		
5–10	2.71	120	1.65		
more than 10 years	2.65	51	1.74		
altogether	2.70	493	1.76		
48. IT BRINGS ME RECOGNITION					
less than 3 years of running training	3.17	148	2.01	0.62	0.605
3–5	3.14	174	1.91		
5–10	3.39	120	1.89		
more than 10 years	3.02	51	1.83		
altogether	3.20	493	1.93		
49. TO HAVE TIME ALONE WITH THE WORLD					
less than 3 years of running training	4.09	148	2.11	0.86	0.463
3–5	4.15	174	1.99		
5–10	4.46	120	1.88		
more than 10 years	4.22	51	1.91		
altogether	4.21	493	1.99		
50. TO GET AWAY FROM IT ALL					
less than 3 years of running training	4.25	148	2.11	1.58	0.195
3–5	4.61	174	1.84		
5–10	4.75	120	1.75		
more than 10 years	4.49	51	2.02		
altogether	4.52	493	1.93		
51. TO MAKE MY BODY PERFORM BETTER THAN BEFORE					
less than 3 years of running training	5.01	148	1.86	0.83	0.478
3–5	4.97	174	1.64		
5–10	5.21	120	1.60		
more than 10 years	4.80	51	1.61		
altogether	5.02	493	1.70		
52. TO BEAT SOMEONE I HAVE NEVER BEATEN BEFORE					
less than 3 years of running training	2.40	148	1.85	2.52	0.058
3–5	2.59	174	1.82		
5–10	2.98	120	1.91		
more than 10 years	2.39	51	1.80		
altogether	2.61	493	1.86		
53. TO FEEL MENTALLY IN CONTROL OF MY BODY					
less than 3 years of running training	4.57	148	2.04	1.23	0.301
3–5	4.60	174	1.84		
5–10	4.85	120	1.76		
more than 10 years	4.31	51	1.75		
altogether	4.62	493	1.88		
54. TO GET COMPLIMENTS FROM OTHERS					
less than 3 years of running training	2.40	148	1.70	0.27	0.848
3–5	2.51	174	1.63		
5–10	2.35	120	1.51		
more than 10 years	2.39	51	1.69		
altogether	2.43	493	1.62		
55. TO FEEL AT PEACE WITH THE WORLD					
less than 3 years of running training	2.99	148	2.00	0.47	0.706
3–5	3.24	174	1.90		
5–10	3.19	120	1.79		
more than 10 years	3.18	51	2.03		
altogether	3.15	493	1.92		
56. TO FEEL LIKE A WINNER					
less than 3 years of running training	4.15	148	2.16	0.16	0.920
3–5	4.31	174	1.98		
5–10	4.24	120	2.10		
more than 10 years	4.22	51	2.02		
altogether	4.24	493	2.06		
